# LncRNA MSC-AS1 aggravates nasopharyngeal carcinoma progression by targeting miR-524-5p/nuclear receptor subfamily 4 group A member 2 (NR4A2)

**DOI:** 10.1186/s12935-020-01202-1

**Published:** 2020-04-28

**Authors:** Hongchao Yao, Like Yang, Linli Tian, Yan Guo, Yushan Li

**Affiliations:** Department of Otolaryngology Head and Neck Surgery, The Second Affiliated Hospital of Harbin Medical University, 246 Xue Fu Road, Harbin, 150086 China

**Keywords:** MSC-AS1, miR-524-5p, NR4A2, NPC

## Abstract

**Background:**

Nasopharyngeal carcinoma (NPC) is a subtype of head and neck cancer with dismal prognosis and high relapse rate. The role of long non-coding RNAs (lncRNAs) in NPC has become a research hotspot in recent years. This study aimed to interrogate the function and mechanism of lncRNA MSC antisense RNA 1 (MSC-AS1) in NPC.

**Methods:**

MSC-AS1 level in NPC tissues and cells were detected by RT-qPCR. Function of MSC-AS1 in NPC cells was assessed by CCK-8, EdU, TUNEL, caspase-3 activity, and transwell invasion assay. Interaction of microRNA-524-5p (miR-524-5p) with MSC-AS1 and nuclear receptor subfamily 4 group A member 2 (NR4A2) was determined by RIP and luciferase reporter assays.

**Results:**

MSC-AS1 was upregulated in NPC tissues and cells. Functional assays indicated that MSC-AS1 exacerbated cell proliferation, hindered apoptosis, and facilitated invasion and epithelial-to-mesenchymal transition (EMT) in NPC. Mechanistically, MSC-AS1 sequestered miR-524-5p to upregulate NR4A2 expression in NPC cells. Finally, NR4A2 was conformed as an oncogene in NPC, and overexpressed NR4A2 could restore MSC-AS1 knockdown-mediated inhibition on NPC progression.

**Conclusions:**

Our study firstly showed that lncRNA MSC-AS1 aggravated NPC progression by sponging miR-524-5p to increase NR4A2 expression, indicating MSC-AS1 as a novel target for the lncRNA-targeted therapy in NPC.

## Background

Nasopharyngeal carcinoma (NPC) is an aggressive type of head and neck malignancy arising from nasopharyngeal epithelium [1]. Currently, NPC patients are still suffering from the unsatisfactory 5-year survival rate which is around 50–70% [2]. In particular, incidence of NPC is the highest in China, and NPC patients in China are becoming younger in average age [3]. NPC progression is known to be deeply associated with genetic and epigenetic abnormalities [4]. Hence, better elucidating of molecular mechanism underlying NPC development is helpful to explore the potential strategies for NPC patients.

Long non-coding RNAs (lncRNAs) are characterized as a set of non-coding RNAs (ncRNAs) with more than 200 nucleotides in length [5]. Large numbers of studies have delineated that lncRNAs function as oncogenes or tumor suppressors in human cancer development [6]. Furthermore, numerous lncRNAs have been reported in NPC progression. For example, lncRNA AFAP1-AS1 regulated miR-423-5p/Rho/Rac axis to aggravate NPC metastasis [7]. LncRNA UCA1 aggravated proliferation, migration and invasion of NPC cells through regulating miR-145 [8]. In this study, we aimed to explore the role of a novel functional lncRNA in NPC. LncRNA MSC antisense RNA 1 (MSC-AS1), with a length of 4802 bp, was reported to indicate poor recurrence-free survival in hepatocellular carcinoma (HCC) [9], function as a tumor facilitator in pancreatic cancer [10], and regulate osteogenic differentiation [11]. Through GEPIA (http://gepia.cancer-pku.cn/), lncRNA MSC-AS1 was highly expressed in head and neck squamous cell carcinoma (HNSC) samples and associated with poor overall survival of patients. Owing to NPC is a kind of head and neck cancer [7, 12, 13], we explored whether MSC-AS1 is associated with the progression of NPC.

MicroRNAs (miRNAs) are another group of ncRNAs with about 22 nucleotides. MiRNAs are recognized as the essential modulators of gene expression by base-pairing to mRNAs 14]. Researchers have indicated the correlation of miRNAs with NPC progression [15, 16]. Notably, plentiful reports have demonstrated the competitive endogenous RNA (ceRNA) mechanism in NPC. The ceRNA mechanism is that lncRNAs function as miRNA sponges to increase the expression of miRNAs’ downstream genes. For example, lncRNA PCAT7 upregulated ELF2 by sponging miR-134-5p to promote tumor growth in NPC [17]; lncRNA SNHG7 regulated miR-145a-5p/NUAK1 axis to drive metastasis and invasion in NPC [18]. In our current study, bioinformatics analysis and RT-qPCR examination revealed the highest binding potential of miRNA with MSC-AS1. MiR-524-5p, with a length of 22 nucleotides, has been reported to possess anti-tumor function in multiple cancers, such as pituitary tumor [19], gastric cancer [20], melanoma [21] and glioma [22]. However, its interaction with MSC-AS1 in NPC has never been explored. In this study, we demonstrated the interaction between MSC-AS1 and miR-524-5p through mechanism investigation and functions of miR-524-5p was disclosed by gain-of-function assays.

Nuclear receptor subfamily 4 group A member 2 (NR4A2) belongs to the NR subfamily 4 group A (NR4A) family which is characterized as a group of immediate-early genes activated by growth factors, mitogens, or other stimuli [23]. Accumulating researches stated that NR4A2 played an oncogenic role in cancers [24–27]. For example, NR4A2 suppressed p53 transactivation to save cells from p53-induced apoptosis [28]. NR4A2 upregulation induced cell growth in cervical cancer and activation of Notch signaling silenced NR4A2 to repress cervical cancer progression [29]. In NPC, it has been reported that high cytoplasmic NR4A2 expression predicted poor prognosis in NPC [30]. However, association of NR4A2 with MSC-AS1 and miR-524-5p has never been revealed in NPC.

Therefore, the purpose of this study is to uncover the impact and mechanism of MSC-AS1 in NPC.

## Materials and methods

### Tissue collection

Between June 2013 and April 2018, amount to 34 pairs of NPC tissues and pair-matched adjacent normal tissues were collected from patients who underwent surgical resection at The Second Affiliated Hospital of Harbin Medical University. Before surgery, patients who received radiotherapy and chemotherapy were excluded. After resection, the coupled tissues were immediately preserved at -80 °C. Ethical approval was obtained from the ethics committee of The Second Affiliated Hospital of Harbin Medical University. All patients for participation provided informed consent.

### Cell culture

Two nasopharyngeal epithelial cells (NP69 and NP460) and four nasopharyngeal carcinoma (NPC) cells (SUNE1, CNE-2, 5-8F, CNE-1) were obtained from the Chinese Academy of Sciences (Shanghai, China). All cells were incubated in RPMI-1640 medium (Gibco, Rockville, MD, USA) containing 10% fetal bovine serum (FBS; Gibco). Cells were incubated under the standard environment (37 °C, 5% CO_2_).

### Cell transfection

At 1day prior to transfection, 5-8F or CNE-1 cells were incubated in six-well plates. Then, cells were transfected via Lipofectamine 2000 (Invitrogen, Carlsbad, CA, USA). Short hairpin RNA (shRNA) targeting MSC-AS1 (sh-MSC-AS1#1/2) and the negative control (sh-NC), miR-23b-3p mimic, miR-524-5p mimic/inhibitor, NC mimic/inhibitor, along with the overexpression of NR4A2, MSC-AS1 and pcDNA3.1 empty vector were constructed from RiboBio (Guangzhou, China). Transfection took place after 48 h, and cells were all harvested. The sequences were shown in Additional file 1: Table S1.

### RNA extraction and RT-qPCR analysis

Total RNA was extracted using TRIzol reagent (Invitrogen), and then the PrimeScript reverse transcriptase reagent kit (Takara, Kusatsu, Japan) was applied to synthesize cDNA. Real-time PCR amplification was then performed using an SYBR Green Real-Time PCR Kit (Applied Biosystems, Foster City, CA, USA) on the Bio-Rad CFX96 System (Applied Biosystems). As the endogenous control, U6/GAPDH was used. The 2^-ΔΔCT^ method was selected for transcript quantification. The primers were listed in Additional file 1: Table S1.

### CCK-8 assay

5-8F or CNE-1 cells were seeded in 96-well plates and incubated at different periods. Each well in fresh medium was added by 10 µl of CCK-8 solution and incubated for 4 h continually. Finally, the absorbance at OD value (450 nm) was assessed through a microplate reader (Bio-Rad, Hercules, CA, USA).

### Colony formation assay

Transfected 5-8F or CNE-1 cells were placed into six-well plates and maintained in medium added with 10% FBS (Gibco). Rinsed through ice-cold PBS (Sigma-Aldrich, St. Louis, MO, USA), colonies were subsequently fixed through methanol (Sigma-Aldrich), dyed using crystal violet (Sigma-Aldrich) and then counted manually.

### EdU assay

5 × 10^4^ transfected cells of 5-8F or CNE-1 were plated into the each well of 96-well plates, and then 100 μL of EdU medium was added for 3 h. The proliferative cells were detected using EdU assay kit (Ribobio) as per instruction. Cells were fixed by 4% paraformaldehyde, permeabilized by 0.5% Troxin X-100 and incubated in 1 × Apollo^®^ 488 staining solution. After DAPI staining (0.3 mM; Sigma-Aldrich) for nuclear detection, cell samples were assayed using fluorescence microscope (Leica, Mannheim, Germany).

### Caspase-3 activity assay

Using Beyotime C1115 Caspase 3 Activity Assay Kit (Beyotime, Nantong, China), the activity of caspase-3 was determined. Isolated proteins from transfected 5-8F or CNE-1 cells were added into 96-well plates with the reaction buffer (Invitrogen). Finally, caspase-3 activity was examined through a microplate reader (Thermo Fisher Scientific) at 405 nm.

### Tunel assay

Transfected 5-8F or CNE-1 cells were incubated with fluorescein-labelled dUTP (Thermo Fisher Scientific). After that, the nucleus was stained by 0.3 mM of DAPI from Sigma-Aldrich. Through the fluorescence microscope (Leica, Mannheim, Germany), apoptotic cells were eventually analyzed.

### Transwell assay

In serum-free medium, transfected 5-8F or CNE-1 cells were planted in the top transwell chambers (Corning Incorporated, Corning, NY, USA), which was coated with Matrigel (BD Biosciences, Franklin Lakes, NJ, USA). The bottom chambers (Corning Incorporated) were plated with medium containing 10% FBS (Gibco). Thereafter, invaded cells were fixed, stained with 1% crystal violet and eventually evaluated through an inverted microscope (magnification: 200 × ; Nikon, Tokyo, Japan).

### Immunofluorescence (IF) assay

Cell samples of 5-8F and CNE-1 on culture slides were incubated for 24 h until adhered to the slides. After that, cells were washed in PBS, fixed by ice-cold methanol for 10 min and blocked in 5% BSA for 10 min. The primary antibodies against E-cadherin and N-cadherin, as well as the secondary antibodies were utilized for the incubation with cells. Following DAPI staining (0.3 mM), cells were observed using fluorescence microscope (Leica).

### Western blotting

Total protein samples from 5-8F or CNE-1 cells were extracted in RIPA lysis buffer, and then separated by electrophoresis on 12% SDS-PAGE (Bio-Rad Laboratories, Hercules, CA, USA). Protein samples were then transferred onto PVDF membranes (Bio-Rad Laboratories) and incubated in 5% nonfat milk for blocking membranes. The diluted primary antibodies (1: 2000; Abcam, Cambridge, MA), including mouse monoclonal anti-E-cadherin antibody (ab76055), rabbit monoclonal anti-N-cadherin antibody (ab202030), mouse monoclonal anti-NR4A2 antibody (ab41917) and mouse monoclonal anti-GAPDH antibody (ab8245) as loading control, were used to probe membranes all night at 4 °C. Following washing in Tris-buffered saline containing 0.1% Tween-20 (TBST), the horse-radish peroxidase (HRP)-tagged rabbit monoclonal secondary antibodies (1: 5000; Abcam) against IgG were added for 2 h at room temperature. At length, the protein blots were observed using ECL detection system (Santa Cruz, CA, USA) in the dark, and then placed into gel imager, followed by analysis of Bio-Rad image analysis system (Bio-Rad). The gray values of the target proteins and the internal control were compared employing the Image J software (NIH, Bethesda, MD, USA).

### Bioinformatics analysis methods

Expression profile of MSC-AS1 in HNSC samples and its association with overall survival of HNSC patients were downloaded from GEPIA (http://gepia.cancer-pku.cn/) [31]. The binding sequences between RNAs were predicted from starBase v2.0 (http://starbase.sysu.edu.cn/) [32].

### Subcellular fractionation

Using the nuclear/cytoplasmic Isolation Kit (Biovision, San Francisco, CA, USA), subcellular fractionation in 5-8F or CNE-1 cells was carried out as instructed. Cells were initially lysed in the cell fractionation buffer, and centrifuged for the separation of cytosolic and nuclear fractions. The supernatant was transferred into a fresh RNase-free tube. Thereafter, the remaining lysates were rinsed in cell fractionation buffer and centrifuged. The cell disruption buffer was added for lysing cell nuclei. Lysates and the supernatant were mixed in 2 × lysis/binding solution, equal volume of ethanol was added. After washing, TRIzol reagent was added to isolate RNAs. Finally, the expression ratios of MSC-AS1 were determined by RT-qPCR, with U6 and GAPDH used as the control for nuclear RNA and cytoplasmic RNA.

### RNA FISH

The specific RNA FISH probe to MSC-AS1 was synthesized by Ribobio Company. Cells of CNE-1 and 5-8F were fixed by 4% formalin, treated with protease K at 37 °C, and then rinsed in PBS. Cells were dehydrated in ethanol. 20 μl of hybridization solution containing 2 μl of probe and 18 μl of pre–hybridization solution was prepared to incubate with cells all night at 42 °C. Afterwards, cells were rinsed twice in 25% formamide/2 × saline sodium citrate (SSC). DAPI solution (0.3 mM) was then added for nuclear counter-staining. Samples were observed under fluorescence microscope.

### Microarray

54 miRNAs potentially bound to MSC-AS1 were obtained from starBase. The expressions of 54 miRNAs were detected by PCR in three pairs of adjacent normal tissues and NPC tissues. Differentially expressed miRNAs (P < 0.05 and fold change > 2.0) were picked for follow-up analyses, among which miR-524-5p was most significantly down-regulated.

### RNA immunoprecipitation (RIP) Assay

Using a Magna RIP™ RNA Binding Protein Immunoprecipitation Kit (Millipore, Bedford, MA, USA), RIP assay was carried out. After being harvested, transfected 5-8F or CNE-1 cells were lysed in RIP lysis buffer (Solarbio, Beijing, China). Subsequently, magnetic beads (Invitrogen) were added to the RIP lysis buffer (Solarbio) and then conjugated overnight with anti-Ago2 (Abcam) or anti-IgG (Abcam) at 4 °C. The immunoprecipitated RNA was acquired after digestion with proteinase K (Absin, Shanghai, China) and quantified by RT-qPCR.

### Dual-luciferase reporter assay

The wild-type or mutant binding sequence of miR-524-5p in MSC-AS1 or NR4A2 3′-UTR was synthesized and sub-cloned into pmirGLO dual-luciferase vector (Promega, Madison, WI, USA). MSC-AS1-Wt/Mut vector or NR4A2-Wt/Mut vector was co-transfected with NC mimic or miR-524-5p mimic into 5-8F, CNE-1 or 293T cells. 48 h later, the Dual Luciferase Report Assay System (Promega) was employed to monitor luciferase activity.

### RNA pull‐down assay

MiR-524-5p no-biotin probe and miR-524-5p biotin probe were synthesized by Thermo Fisher Scientific. The biotinylated miRNA was incubated with cell lysates (Invitrogen) overnight, followed by adding streptavidin magnetic beads (Invitrogen). Finally, RT-qPCR was used to detect the expression levels.

### Statistical analysis

Data was expressed as mean ± SD. In order to analyze data, SPSS 20.0 software (SPSS Inc., Chicago, IL, USA) was adopted. With the use of Student’s *t* test or one-way ANOVA, group difference was estimated. Besides, P < 0.05 was defined to be statistically significant. All experiments were repeated at least three times. Correlation among NR4A2, MSC-AS1 and miR-524-5p was analyzed via Pearson’s correlation coefficient analysis.

## Results

### MSC-AS1 was upregulated in NPC, silence of MSC-AS1 controlled proliferation and activated apoptosis of NPC cells

**Fig. 1:**
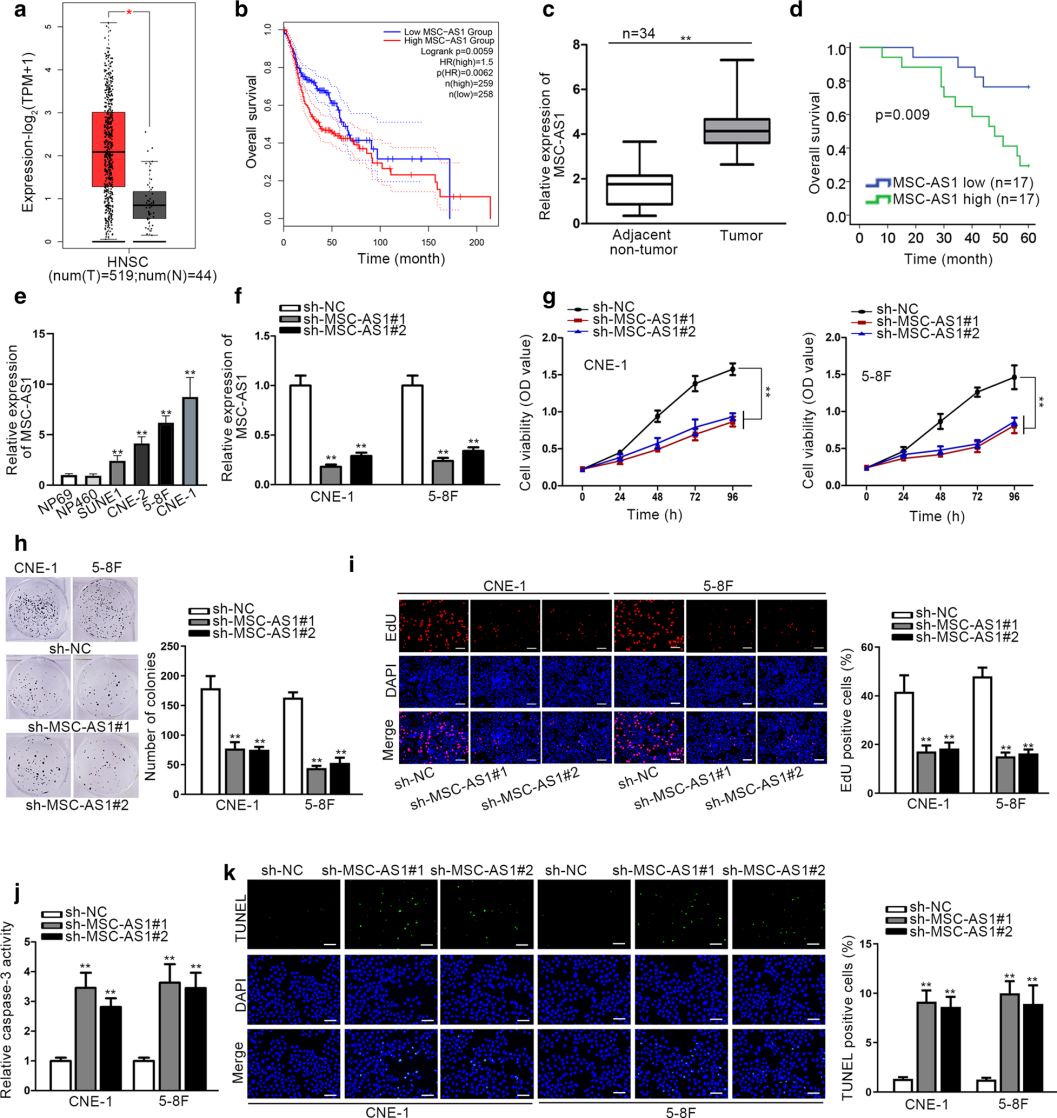
MSC-AS1 was upregulated in NPC, silence of MSC-AS1 controlled proliferation and activated apoptosis in NPC cells. **a** Expression profile of MSC-AS1 in HNSC samples or adjacent non-tumor samples in GEPIA database. **b** Survival analysis of HNSC patients with high or low MSC-AS1 expression in GEPIA database. **c** RT-qPCR data of MSC-AS1 level in NPC tissues and adjacent non-tumorous tissues. **d** Overall survival of patients with high or low MSC-AS1 expression enrolled in this study was analyzed with Kaplan–Meier method. **e** RT-qPCR results of MSC-AS1 level in NPC cells and two nasopharyngeal epithelial cells. **f** RT-qPCR confirmed the knockdown efficiency of MSC-AS1 in CNE-1 and 5-8F cells. (**g**–**i**) The data of CCK-8, colony formation and EdU (scale bar = 200 μm) showed the proliferation of CNE-1 and 5-8F cells transfected with sh-NC, sh-MSC-AS1#1, or sh-MSC-AS1#2. (**j**–**k**) Apoptosis of CNE-1 and 5-8F cells under MSC-AS1 silence was detected by caspase-3 activity and TUNEL staining (scale bar = 200 μm). ^*^P < 0.05, ^**^P < 0.01

Through GEPIA, MSC-AS1 was identified as a highly-expressed lncRNA in HNSC samples (Fig. 1a). Besides, MSC-AS1 presented an association with the overall survival rate in HNSC, which included NPC (Fig. 1b). Thus, we further researched the association of MSC-AS1 with NPC. Firstly, the expression profile of MSC-AS1 was determined in NPC samples. We found that MSC-AS1 level was elevated in NPC samples compared with the paired adjacent non-tumor samples (Fig. 1c). Based on the cut-off value (median value) of MSC-AS1 expression in 34 patient samples, the overall survival of patients with high or low MSC-AS1 level was analyzed. As indicated in Fig. 1d, high level of MSC-AS1 was associated with the low overall survival of NPC patients. In addition, MSC-AS1 expression was higher in NPC cells than in nasopharyngeal epithelial cells (Fig. 1e). Then, the function of MSC-AS1 in NPC was determined through loss-of-function assays. Since CNE-1 and 5-8F cells were validated to present the higher MSC-AS1 level, we silenced MSC-AS1 level in these two cells by sh-MSC-AS1#1/2, and the transfection efficiency was confirmed by RT-qPCR (Fig. 1f). Thereafter, we performed CCK-8, colony formation and EdU assays to explore the function of MSC-AS1 knockdown on NPC cell proliferation. As a result, silenced MSC-AS1 significantly inhibited the proliferation of two NPC cells (Fig. 1g–i). Besides, the apoptosis of NPC cells was detected by caspase-3 activity and TUNEL staining. Results showed that knockdown of MSC-AS1 induced caspase-3 activity and increased the TUNEL staining ratio in NPC cells (Fig. 1j–k), indicating that MSC-AS1 depletion prompted apoptosis in NPC cells. Above results confirmed that MSC-AS1 was upregulated in NPC tissues and cells, promoted cell proliferation and refrained cell apoptosis.

### MSC-AS1 acted as an oncogene in NPC cells

**Fig. 2:**
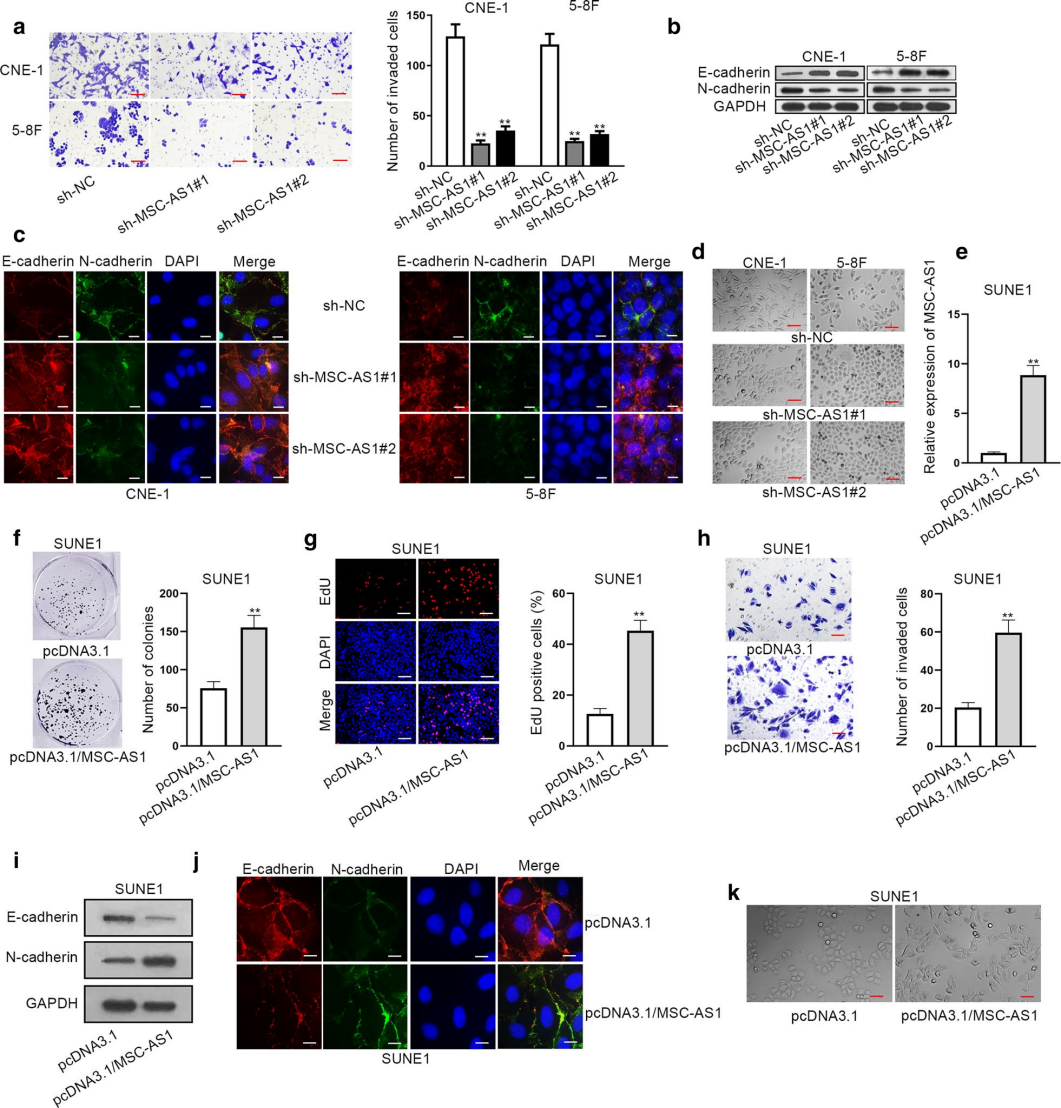
MSC-AS1 played oncogenic role in NPC cells. **a** Images and quantification of invaded CNE-1 and 5-8F cells transfected with sh-NC, sh-MSC-AS1#1, or sh-MSC-AS1#2 was obtained through transwell assay (scale bar = 200 μm). (**b**–**c**) Western blot results and IF (scale bar = 50 μm) staining pictures showed E-cadherin and N-cadherin levels in CNE-1 and 5-8F cells under MSC-AS1 silence. **d** Morphology of cells transfected with sh-NC, sh-MSC-AS1#1/2 was shown. Scale bar = 200 μm. **e** Overexpression of MSC-AS1 with pcDNA3.1/MSC-AS1 vector. **f–g** Colony formation and EdU assays (scale bar = 200 μm) assessed the proliferation in MSC-AS1-overexpressed NPC cells. **h** Transwell invasion assay (scale bar = 200 μm) was conducted to test cell invasion after overexpression of MSC-AS1. (**i–j**) EMT markers were assessed with western blot analysis and IF (scale bar = 50 μm) in pcDNA3.1 and pcDNA3.1/MSC-AS1 groups. **k** Morphology of SNUE1 cell was observed with the transfection of pcDNA3.1 and pcDNA3.1/MSC-AS1. Scale bar = 200 μm. ^**^P < 0.01

Later, we detected the effect of MSC-AS1 on invasive capacity of NPC cells. Transwell assay depicted that the number of invaded NPC cells was decreased upon the depletion of MSC-AS1 (Fig. 2a). Moreover, EMT progression was evaluated by detecting the levels of epithelial and mesenchymal markers. As shown, E-cadherin protein level was boosted, whereas N-cadherin protein level was reduced responding to MSC-AS1 knockdown in NPC cells (Fig. 2b). Accordingly, the same results were observed in IF assay (Fig. 2c). Moreover, morphology of cells was observed after MSC-AS1 was silenced. It was found that silencing of MSC-AS1 inhibited the EMT phenotype in two NPC cells (Fig. 2d). Hence, it was suggested that knockdown of MSC-AS1 hindered cell invasion and EMT in NPC. To further validate the oncogenic role of MSC-AS1 in NPC, gain-of-function assays were carried out in SUNE1 cells which presented a relative lowest level of MSC-AS1. At first, MSC-AS1 was overexpressed in SUNE1 cells (Fig. 2e). Colony formation assay and EdU assay indicated that cell proliferation was promoted after overexpression of MSC-AS1 (Fig. 2f–g). Additionally, enhanced level of MSC-AS1 aggravated cell invasion (Fig. 2h) and EMT process (Fig. 2i–k). Altogether, it was indicated that MSC-AS1 functioned as an oncogene in NPC.

### MSC-AS1 sponged miR-524-5p in NPC cells

**Fig. 3:**
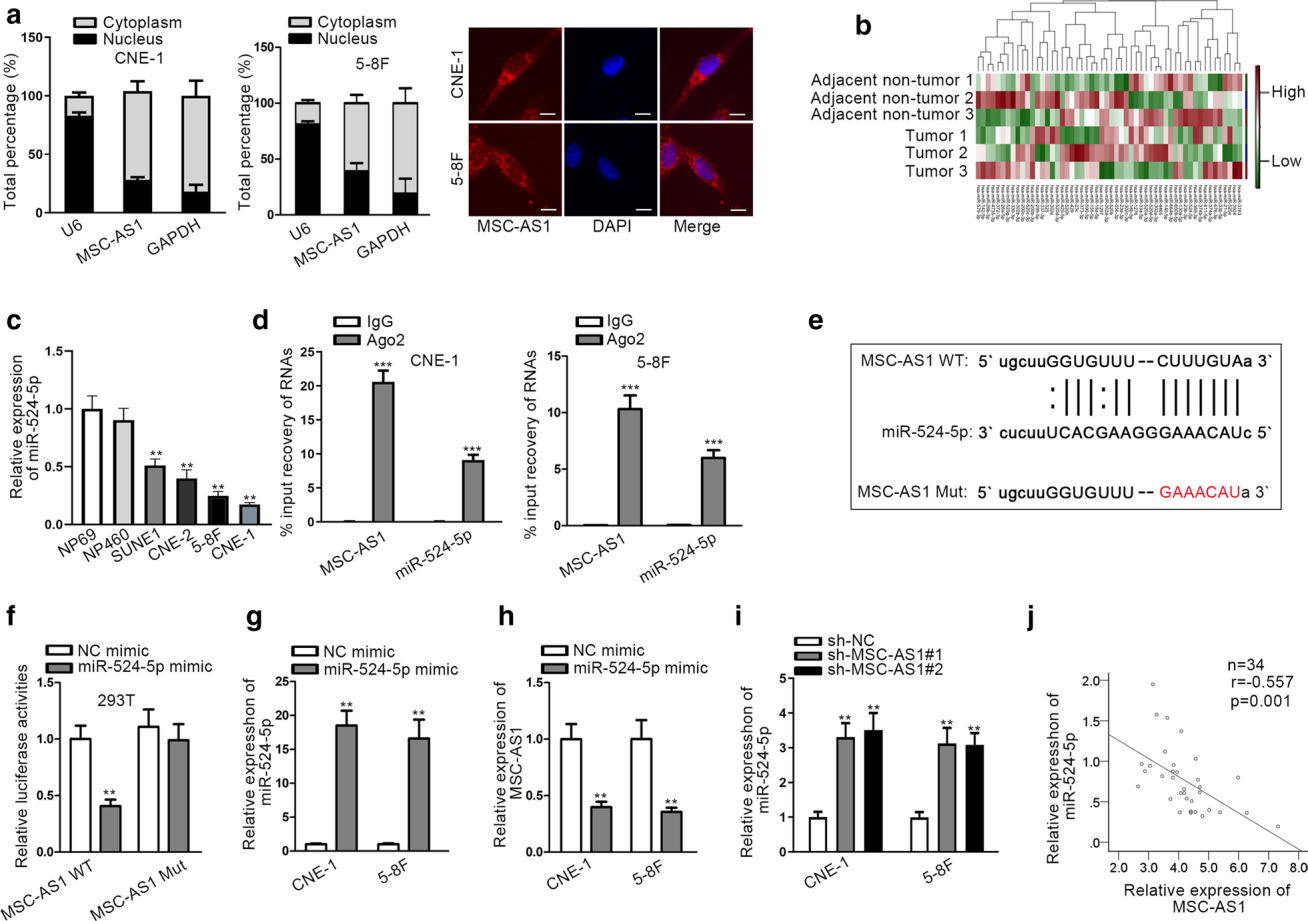
MSC-AS1 sponged miR-524-5p in NPC cells. **a** Subcellular fractionation and FISH (scale bar = 50 μm) showed MSC-AS1 level in cytoplasm and nucleus of CNE-1 and 5-8F cells. **b** Heatmap showed the level of 54 predicted miRNAs in 3 pairs of NPC tissues and adjacent non-tumor tissues. **c** RT-qPCR results of miR-524-5p level in NPC cells and nasopharyngeal epithelial cells. **d** RIP assay was used to determine the interaction between MSC-AS1 and miR-524-5p. **e** The miR-524-5p binding site on MSC-AS1 and designed mutated site were shown. **f** Luciferase reporter assay was applied to detect the interaction between miR-524-5p and MSC-AS1. **g** RT-qPCR results confirmed the overexpression efficiency of miR-524-5p in CNE-1 and 5-8F cells. **h** Expression of MSC-AS1 in CNE-1 and 5-8F cells under miR-524-5p overexpression was detected by RT-qPCR. **i** Expression of miR-524-5p in CNE-1 and 5-8F cells under MSC-AS1 silence was detected by RT-qPCR. **j** Pearson’s correlation analysis showed the negative expression correlation between MSC-AS1 and miR-524-5p in NPC tissues. ^**^P < 0.01, ^***^P < 0.001

The mechanisms of lncRNAs are diverse [33]; it has been reported that lncRNAs could regulate gene expression at transcriptional level in the nucleus [34]. More studies indicated that lncRNAs could function as ceRNAs at post-transcriptional level in the cytoplasm [35]. Through subcellular fractionation assay and FISH assay, we found that MSC-AS1 was mainly expressed in cytoplasm of NPC cells (Fig. 3a), indicating the post-transcriptional regulation of MSC-AS1 in NPC. Thus, the molecular regulatory mechanism that MSC-AS1 acted as a ceRNA in NPC was investigated. We found that 54 candidate miRNAs could bind to MSC-AS1 through browsing starBase v2.0 (http://starbase.sysu.edu.cn/). To detect their implication in NPC, we analyzed the expressions of these miRNAs in NPC tissues. Consequently, miR-524-5p was found as the most significantly downregulated miRNA in NPC tissues compared with matched adjacent non-cancerous tissues (Fig. 3b). Former studies have showed that miR-524-5p was lowly expressed and served as an anti-tumor gene in multiple cancers [19–22]. Therefore, we chose miR-524-5p for further exploration. The downregulation of miR-524-5p in NPC cells was validated as well (Fig. 3c). RIP assay showed that miR-524-5p could be co-immunoprecipitated with MSC-AS1 by Ago2 antibody in NPC cells (Fig. 3d). Besides, we obtained the binding sequence between MSC-AS1 and miR-524-5p, and mutated the sequence on MSC-AS1 for luciferase activity assay (Fig. 3e). Results showed that overexpression of miR-524-5p decreased the luciferase activity of MSC-AS1 WT rather than MSC-AS1 Mut (Fig. 3f). According to the results of heatmap, miR-23b-3p also presented significant downregulation in NPC tissues. Thus, the binding sequence between MSC-AS1 and miR-23b-3p was predicted (Additional file 2: Figure S1a). However, luciferase reporter assay indicated that the luciferase activity of both MSC-AS1 WT and MSC-AS1 Mut was not changed by miR-23b-3p mimic (Additional file 2: Figure S1b). This data suggested that only miR-524-5p was sponged by MSC-AS1 in NPC. Then, we overexpressed miR-524-5p in NPC cells by miR-524-5p mimic (Fig. 3g). RT-qPCR showed that MSC-AS1 level was downregulated in NPC cells transfected with miR-524-5p mimic (Fig. 3h). Afterwards, we found that MSC-AS1 knockdown induced the expression of miR-524-5p in NPC cells (Fig. 3i). The negative expression correlation between MSC-AS1 and miR-524-5p levels in NPC tissues was confirmed as well (Fig. 3j). Next, we also detected the function of miR-524-5p in NPC cellular processes. In response to the upregulation of miR-524-5p, cell growth was suppressed (Additional file 2: Figure: S1c–e) and EMT process was inhibited (Additional file 2: Figure S1f), indicating the tumor-suppressive role of miR-524-5p in NPC. These data indicated that miR-524-5p was sponged by MSC-AS1 and acted as a tumor-suppressor in NPC.

### MSC-AS1 induced NR4A2 level by sponging miR-524-5p in NPC cells

**Fig. 4:**
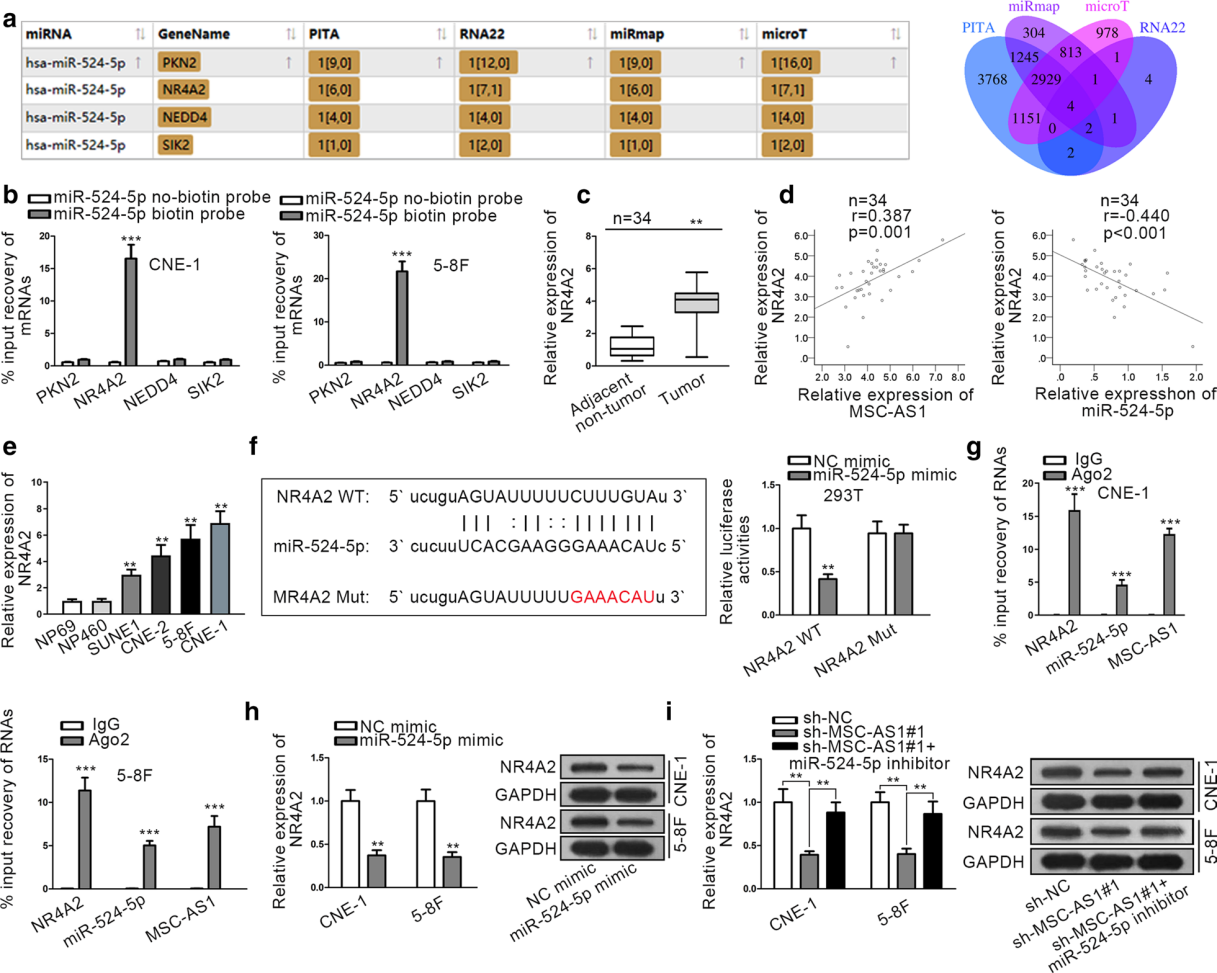
MSC-AS1 induced NR4A2 level by targeting miR-524-5p in NPC cells. **a** The list and Venn pattern showed that PKN2, NR4A2, NEDD4, and SIK2 were potentially targeted by miR-524-5p as predicted by PITA, RNA22, miRmap and microT. **b** RNA pull down assay was used to examine the interaction of miR-524-5p with the predicted mRNAs. **c** RT-qPCR results of NR4A2 expression in NPC tissues and the paired adjacent non-tumor tissues. **d** Pearson’s correlation curve showed that NR4A2 was positively correlated with MSC-AS1 and negatively correlated with miR-524-5p in NPC tissues. **e** Expression of NR4A2 in NPC cells and nasopharyngeal epithelial cells. **f** The miR-524-5p binding site on NR4A2 and designed mutated site. Luciferase reporter assay was used to detect the interaction between miR-524-5p and NR4A2. **g** RIP assay was used to determine the interaction of miR-524-5p with MSC-AS1 and NR4A2. **h** RT-qPCR and western blot results of mRNA and protein levels of NR4A2 in NPC cells under miR-524-5p overexpression. **i** RT-qPCR and western blot results of mRNA and protein levels of NR4A2 in NPC cells with indicated transfections. ^**^P < 0.01, ^***^P < 0.001

Subsequently, we tried to find the target genes of miR-524-5p. As presented in Fig. 4a, the intersection of 4 bioinformatics tools (PITA, RNA22, miRmap, and microT) showed that PKN2, NR4A2, NEDD4, and SIK2 were potential targets of miR-524-5p. To investigate the interaction of miR-524-5p with these 4 mRNAs, RNA pull down assay was carried out. Results showed that only NR4A2 was enriched in miR-524-5p biotin probe while other mRNAs couldn’t be pulled down (Fig. 4b), indicating that NR4A2 interacted with miR-524-5p in NPC cells. NR4A2 was known as a member of NR4A family [23]. Multiple studies have revealed that NR4A2 functioned as a carcinogene in several cancers, and indicated poor prognosis in NPC patients [28–30]. Hence, we deduced that MSC-AS1 might regulate NR4A2 in NPC cells through miR-524-5p. We then confirmed that NR4A2 was upregulated in NPC samples versus the adjacent non-cancerous tissues (Fig. 4c). Also, NR4A2 expression was confirmed to be positively correlated with MSC-AS1 expression and negatively correlated with miR-524-5p expression in NPC samples (Fig. 4d). Moreover, NR4A2 level was higher in NPC cells than nasopharyngeal epithelial cells (Fig. 4e). Thereafter, we detected the interaction between miR-524-5p and NR4A2. The miR-524-5p binding sequence on NR4A2 and the mutated sequence were presented. Furthermore, overexpression of miR-524-5p led to the reduced luciferase activity of NR4A2 WT but not NR4A2 Mut in 293T cells (Fig. 4f). Besides, the enrichments of NR4A2, miR-524-5p, and MSC-AS1 in the precipitates of Ago2 were confirmed by RT-qPCR following the RIP analysis (Fig. 4g). Then, the regulation of MSC-AS1/miR-524-5p axis on NR4A2 level was examined. NR4A2 mRNA and protein levels were reduced by miR-524-5p mimic in NPC cells (Fig. 4h). In addition, the inhibition of miR-524-5p restored the expressions of NR4A2 mRNA and protein reduced in MSC-AS1-silenced NPC cells (Fig. 4i). Jointly, these data implied that MSC-AS1 induced NR4A2 level by sponging miR-524-5p in NPC cells.

### Knockdown of NR4A2 suppressed NPC cell growth and EMT process

**Fig. 5:**
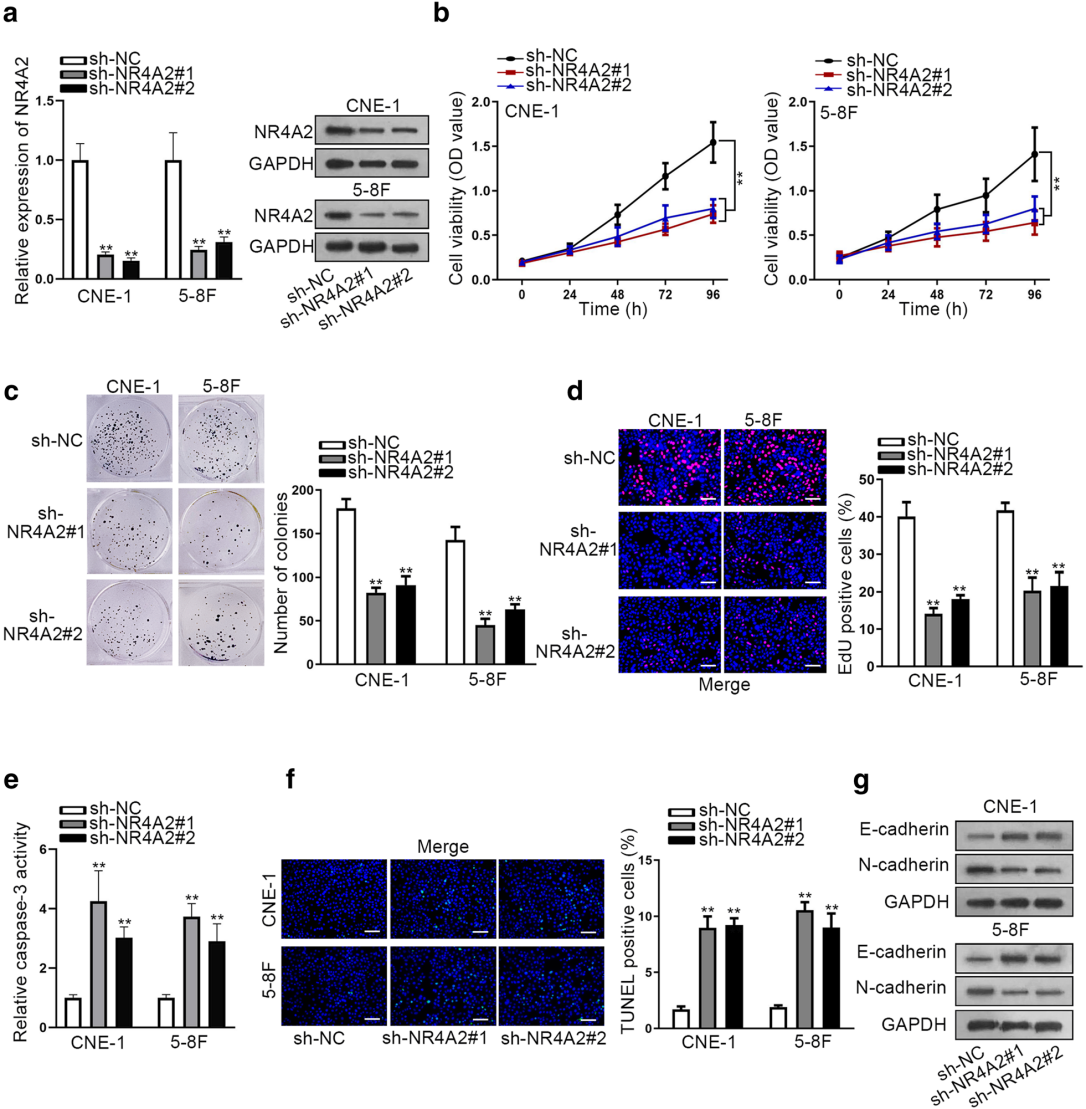
Knockdown of NR4A2 suppressed NPC cell growth and EMT process. **a** NR4A2 was silenced in two NPC cells with specific shRNAs. **b–d** Cell proliferation was monitored after indicated transfections through CCK-8 assay, colony formation assay and EdU assay (scale bar = 200 μm). **e–f** Caspase-3 activity and TUNEL-positive cells in NR4A2-downregulated group and control group were observed. Scale bar = 200 μm. **g** Levels of EMT-relevant proteins in NR4A2 silenced cells were monitored through western blot analysis. ^**^P < 0.01

To further reveal the function of NR4A2 in NPC, we conducted loss-of-function assays in two NPC cells. At first, NR4A2 expression was silenced in CNE-1 and 5-8F cells (Fig. 5a). Cell proliferation was monitored through several proliferation assays. Unsurprisingly, silencing of NR4A2 observably inhibited the proliferative ability of two NPC cells (Fig. 5b–d). Additionally, cell apoptosis was observed in NR4A2-downregulated NPC cells. As indicated in Fig. 5e–f, cell apoptosis was accelerated by the knockdown of NR4A2. Through western blot analysis, we concluded that EMT process was impaired by the silencing of NR4A2 (Fig. 5g). These findings reflected the same function of NR4A2 as MSC-AS1 in NPC cells.

### MSC-AS1 regulated NPC progression by increasing NR4A2 expression

**Fig. 6:**
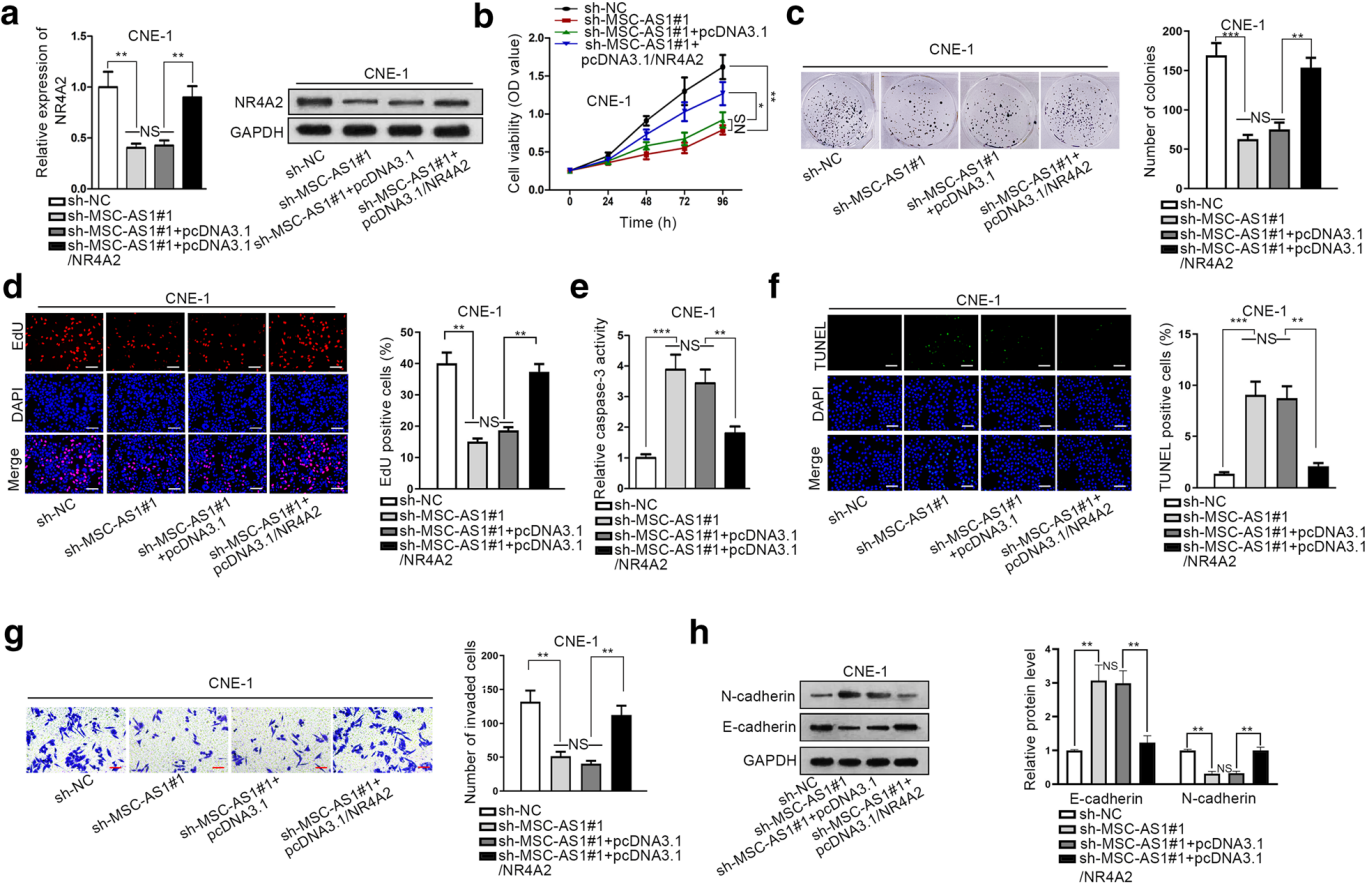
MSC-AS1 regulated NPC progression in a NR4A2-required way. **a** RT-qPCR and western blot results of mRNA and protein levels of NR4A2 in CNE-1 cells under indicated transfections. **b–d** CCK-8, colony formation and EdU (scale bar = 200 μm) showed the proliferation of CNE-1 cells under indicated transfections. **e–f** Caspase-3 activity assay and TUNEL staining (scale bar = 200 μm) were applied to detect apoptosis of CNE-1 cells under indicated transfections. **g** Transwell invasion assay (Scale bar = 200 μm) was implemented to determine the invasion of CNE-1 cells under indicated transfections. **h** Western blot analysis of E-cadherin and N-cadherin expressions in CNE-1 cells under indicated transfections. ^*^P < 0.05, ^**^P < 0.01, ^***^P < 0.001, *NS* presented no significance

To figure out whether MSC-AS1 promoted NPC progression by regulating NR4A2, rescue experiments were implemented in CNE-1 cells. We first confirmed that NR4A2 mRNA and protein levels reduced by MSC-AS1 silence were recovered under the co-transfection of pcDNA3.1/NR4A2 in CNE-1 cells (Fig. 6a). Besides, the prohibitive effect of MSC-AS1 silence on CNE-1 cell proliferation was abrogated by NR4A2 overexpression (Fig. 6b–d). Later, we observed that MSC-AS1 knockdown increased the apoptosis of CNE-1 cells, but such acceleratory effect was reversed by overexpression of NR4A2 (Fig. 6e–f). In addition, the invasive ability of CNE-1 cells retarded by MSC-AS1 silence was recovered by NR4A2 overexpression (Fig. 6g). Subsequently, NR4A2 overexpression reversed the induced level of E-cadherin and reduced level of N-cadherin in NPC cells with MSC-AS1 depletion (Fig. 6h). In conclusion, it was suggested that MSC-AS1 hastened NPC cell proliferation, restrained apoptosis, facilitated invasion and EMT through regulating NR4A2.

## Discussion

The development of NPC was known as a multistep process involving diverse external and internal factors [36]. Evidence has proved that expression alteration of certain lncRNAs is closely correlated with the initiation and development of NPC [37]. Previous reports have uncovered multiple lncRNAs that participate in NPC, regulating cell growth, apoptosis, migration and invasion [7, 8]. Formerly, it was confirmed that high expression of MSC-AS1 indicated low recurrence-free survival of HCC [9], indicating that MSC-AS1 might function as an oncogene in human cancer development. Herein, we found that MSC-AS1 level was upregulated in NPC tissues and cells. Functionally, MSC-AS1 enhanced proliferation, restrained apoptosis, induced invasion and EMT in NPC cells. These findings indicated that MSC-AS1 exerted oncogenic effect in NPC.

Mechanistically, the lncRNA-miRNA-mRNA interaction, which is recognized as ceRNA network, has been widely reported in NPC progression [17, 18]. Herein, we found 54 candidate miRNAs that could be the downstream genes of MSC-AS1. Among which, miR-524-5p was the most significantly downregulated miRNA in NPC, indicating its high potential of association with NPC tissues. Previously, miR-524-5p has been confirmed as a tumor-suppressor in multiple cancers, such as pituitary tumor [19], gastric cancer [20], melanoma [21] and glioma [22]. Accordantly, we confirmed that miR-524-5p was downregulated in NPC tissues and cells, and that MSC-AS1 sponged miR-524-5p in NPC. These indicated that miR-524-5p was an anti-oncogene in NPC.

Moreover, we identified that NR4A2 was a downstream target of miR-524-5p. As a member of NR4A family [23], NR4A2 has been documented as a carcinogene in multiple cancers [28–30]. For example, high NR4A2 expression predicted poor prognosis and promoted chemo-resistance in squamous cell carcinoma (SCC) and colorectal cancer [38, 39]. Also, a former study revealed that upregulation of NR4A2 contributed to poor prognosis in NPC [30]. Concordantly, current study showed that NR4A2 was highly expressed in NPC tissues and cells, and confirmed the interaction between miR-524-5p and NR4A2. Furthermore, it was indicated that MSC-AS1 upregulated NR4A2 through sponging miR-524-5p. Finally, it was found that NR4A2 upregulation rescued the inhibitory proliferation, invasion, EMT and induced apoptosis in MSC-AS1 silenced NPC cells. All above data indicated that MSC-AS1 facilitated NPC progression in NR4A2-dependent way.

## Conclusion

In conclusion, current study firstly unveiled that MSC-AS1 aggravated NPC progression by targeting miR-524-5p/NR4A2 axis, indicating a new insight to the treatment of NPC. However, studies based on animal models and clinical data are required in the future to consolidate the therapeutic value of MSC-AS1 in NPC.

## Supplementary information

**Additional file 1: Table S1.** Sequences for PCR primers and plasmids used in this study.

**Additional file 2: Figure S1.** Upregulation of miR-524-5p hampered NPC cell growth.
