# Supplementary figures and images for: LncRNA MSC-AS1 aggravates nasopharyngeal carcinoma progression by targeting miR-524-5p/nuclear receptor subfamily 4 group A member 2 (NR4A2)
Source: Cancer Cell Int. 2020 Apr 28;20:138. doi: 10.1186/s12935-020-01202-1 (PMC7189691; doi:10.1186/s12935-020-01202-1)

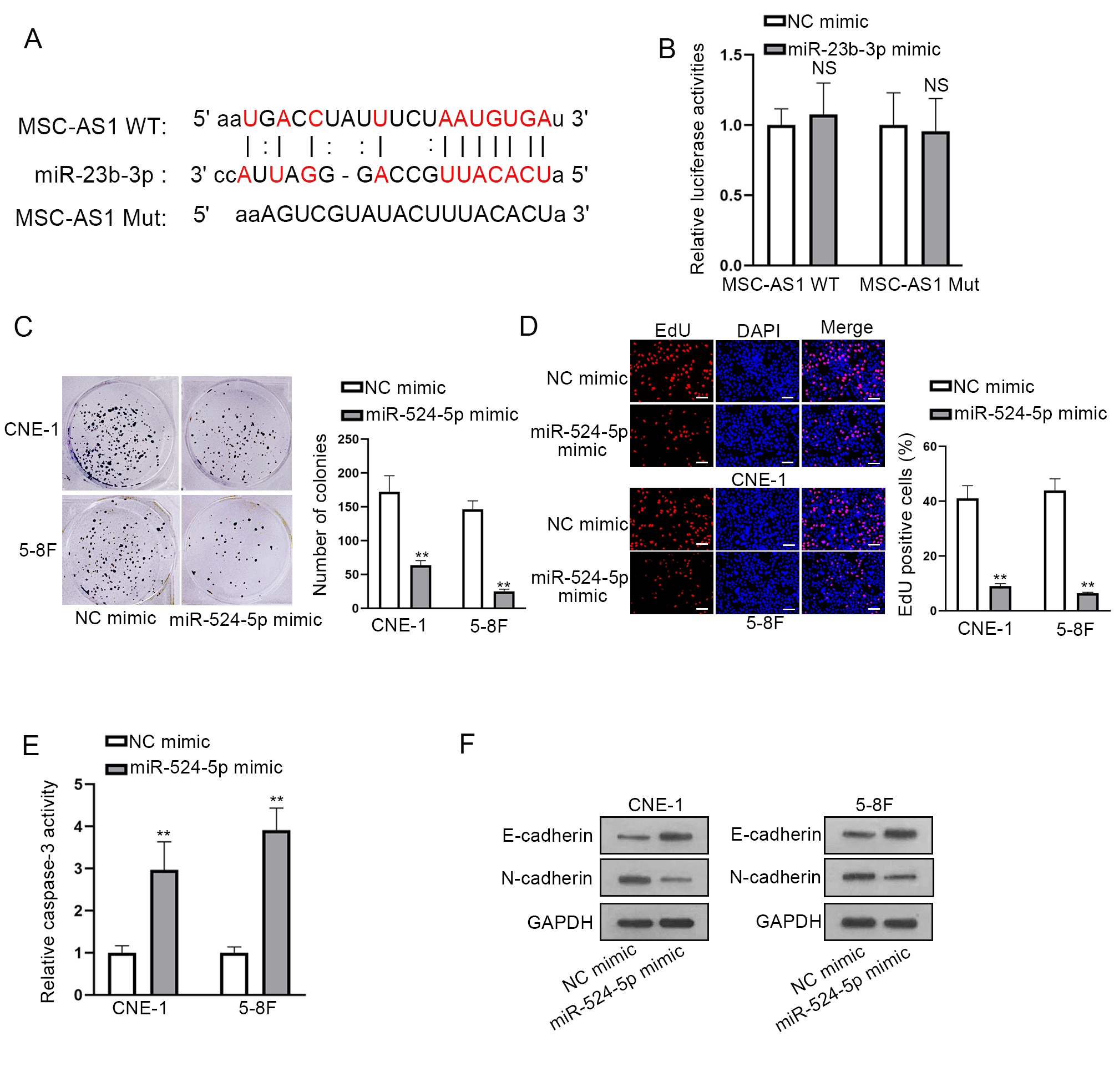

Supplement: Supplementary file 2 — Additional file 2: Figure S1. Upregulation of miR-524-5p hampered NPC cell growth. [file 12935_2020_1202_MOESM2_ESM.tif]
